# Supplementary figures and images for: Detection of disease-specific signatures in B cell repertoires of lymphomas using machine learning
Source: PLoS Comput Biol. 2024 Jul 2;20(7):e1011570. doi: 10.1371/journal.pcbi.1011570 (PMC11249212; doi:10.1371/journal.pcbi.1011570)

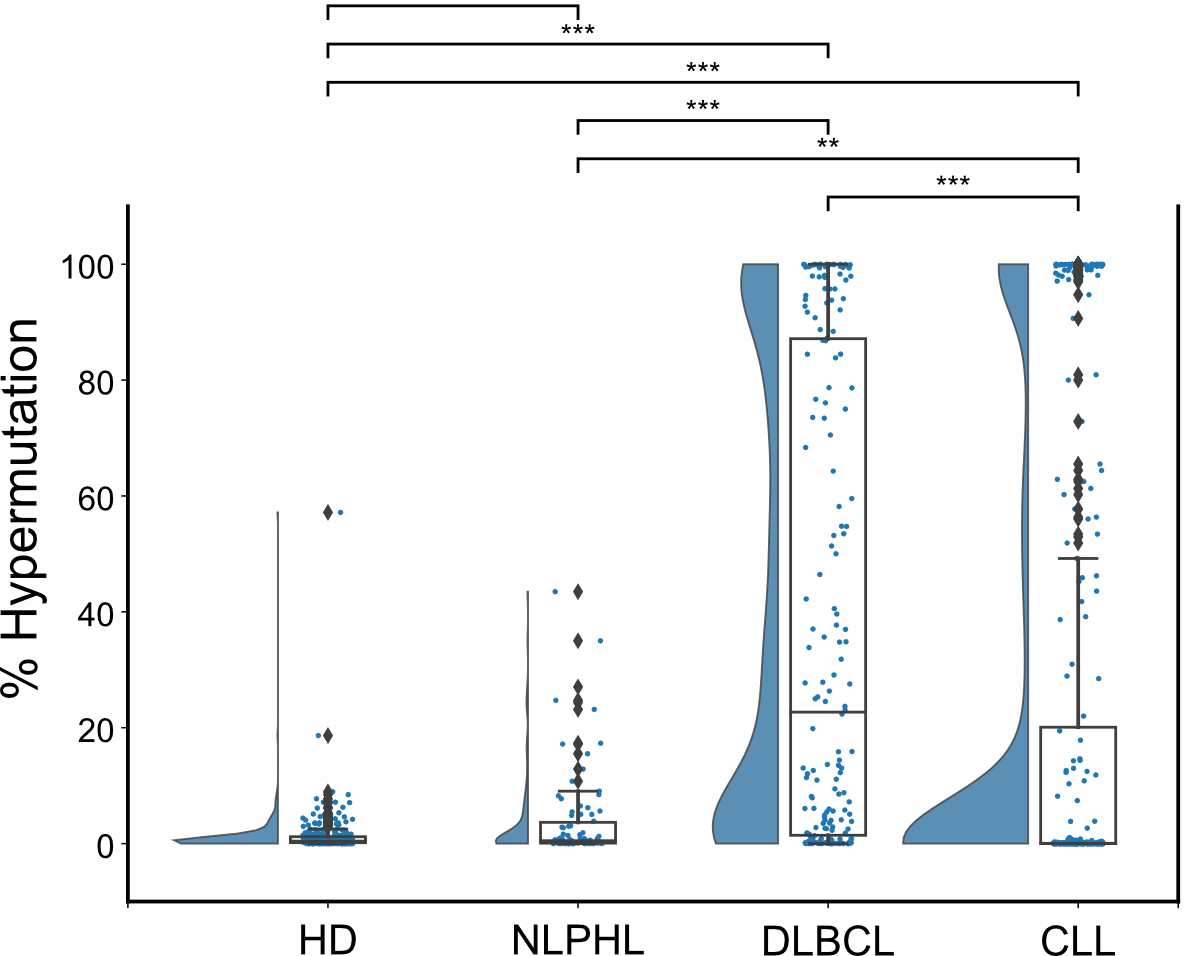

Supplement: S1 Fig — Somatic hypermutation rate calculated based on the 10 most frequent clonotypes per repertoire. (TIFF) [file pcbi.1011570.s001.tiff]

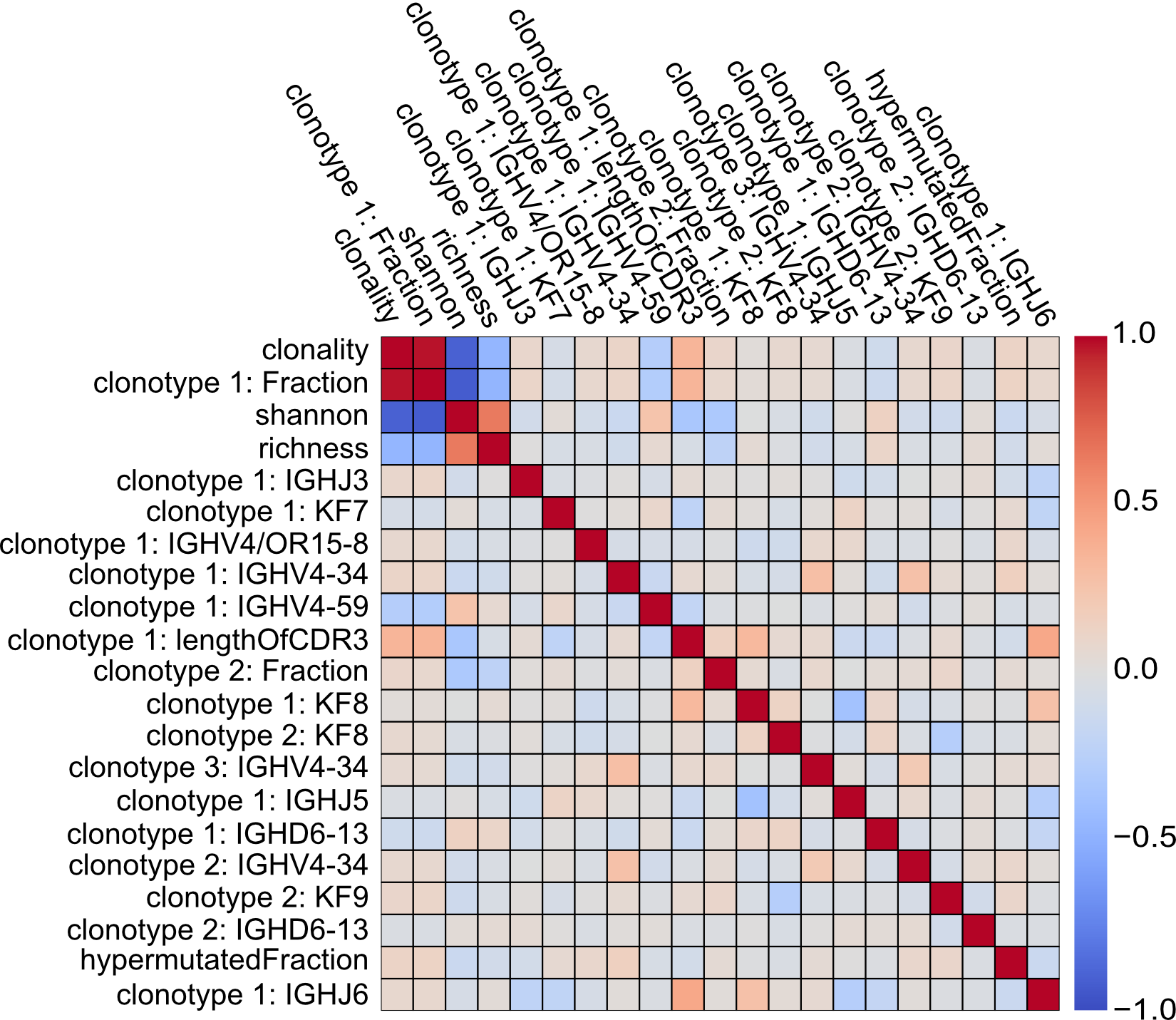

Supplement: S2 Fig — Correlation of major immune repertoire metrics along with strongest predictors from the best performing model for discrimination of HD vs. NLPBL vs. DLBCL vs. CLL. (TIFF) [file pcbi.1011570.s002.tiff]
