# Supplementary material for: Detection of disease-specific signatures in B cell repertoires of lymphomas using machine learning
Source: PLoS Comput Biol. 2024 Jul 2;20(7):e1011570. doi: 10.1371/journal.pcbi.1011570 (PMC11249212; doi:10.1371/journal.pcbi.1011570)
Supplement: S2 Table — (DOCX) [file pcbi.1011570.s004.docx]

**S2 Table. Hyperparameter settings.
Hyperparameters used during training**

|  | Feature | Values |
| --- | --- | --- |
| General | number of clonotypes | 1, 2, 3, 4, 5, 10, 20, 50, 100 |
|  | standardize data | Yes, No |
| Random forest | number of decision trees | 200, 800 |
|  | maximum depth of trees | 8, 16 |
| Logistic Regression | regularization | L2, L1 |
|  | C | [0.01, 0.1, 1, 10] |

**Final Hyperparameters**

| **HD vs. DLBCL vs. CLL** | Feature | Values |
| --- | --- | --- |
| General | number of clonotypes | 3 |
|  | standardize data | Yes |
| Logistic Regression | regularization | L2 |
|  | C | 10 |
| **HD vs. NLPBL vs. DLBCL** | Feature | Values |
| General | number of clonotypes | 20 |
|  | standardize data | Yes |
| Logistic Regression | regularization | L2 |
|  | C | 0.1 |
| **HD vs. NLPBL vs. DLBCL vs. CLL** | Feature | Values |
| General | number of clonotypes | 20 |
|  | standardize data | Yes |
| Logistic Regression | regularization | L2 |
|  | C | 1 |
